# Investigation of Antimicrobial and Cytotoxic Properties and Specification of Silver Nanoparticles (AgNPs) Derived From *Cicer arietinum* L. Green Leaf Extract

**DOI:** 10.3389/fbioe.2022.855136

**Published:** 2022-03-07

**Authors:** Ayşe Baran, Mehmet Fırat Baran, Cumali Keskin, Abdulkerim Hatipoğlu, Ömer Yavuz, Sevgi İrtegün Kandemir, Mehmet Tevfik Adican, Rovshan Khalilov, Afat Mammadova, Elham Ahmadian, Gvozden Rosić, Dragica Selakovic, Aziz Eftekhari

**Affiliations:** ^1^ Department of Biology, Mardin Artuklu University Graduate Education Institute, Mardin, Turkey; ^2^ Department of Medical Services and Techniques, Vocational School of Health Services, Mardin Artuklu University, Mardin, Turkey; ^3^ Joint Ukrainian-Azerbaijan International Research and Education Center of Nanobiotechnology and Functional Nanosystems, Drohobych, Ukraine; ^4^ Department of Nutrition and Dietetics, Faculty of Health Sciences, Mardin Artuklu University, Mardin, Turkey; ^5^ Department of Chemistry, Faculty of Science, Dicle University, Diyarbakir, Turkey; ^6^ Dicle University Central Research Laboratory, , Diyarbakir, Turkey; ^7^ Department of Medical Biology, Dicle University Central Research Laboratory, Faculty of Medicine, Dicle University, Diyarbakir, Turkey; ^8^ Electricity and Energy Department, Vocational School, Mardin Artuklu University, Mardin, Turkey; ^9^ Department of Biophysics and Biochemistry, Baku State University, Baku, Azerbaijan; ^10^ Institute of Radiation Problems, National Academy of Sciences of Azerbaijan, Baku, Azerbaijan; ^11^ Russian Institute for Advanced Study, Moscow State Pedagogical University, Moscow, Russia; ^12^ Department of Botany and Plant Physiology, Baku State University, Baku, Azerbaijan; ^13^ Kidney Research Center, Tabriz University of Medical Sciences, Tabriz, Iran; ^14^ Department of Physiology, Faculty of Medical Sciences, University of Kragujevac, Kragujevac, Serbia; ^15^ Health Innovation & Accelerations Center, Tabriz University of Medical Sciences, Tabriz, Iran; ^16^ Stem Cell Research Center, Tabriz University of Medical Sciences, Tabriz, Iran

**Keywords:** cytotoxic activity, green synthesis, nanomaterials, food pathogens, nanomedicine, SEM-EDX

## Abstract

Using biological materials to synthesize metallic nanoparticles has become a frequently preferred method by researchers. This synthesis method is both fast and inexpensive. In this study, an aqueous extract obtained from chickpea (*Cicer arietinum* L.) (CA) leaves was used in order to synthesize silver nanoparticles (AgNPs). For specification of the synthesized AgNPs, UV-vis spectrophotometer, Fourier transform infrared spectroscopy (FT-IR), X-ray diffraction analysis (XRD), transmission electron microscopy (TEM), scanning electron microscopy (SEM), electron dispersive X-ray (EDX), and zeta potential (ZP) analyses data were used. Biologically synthesized AgNPs demonstrated a maximum surface plasmon resonance of 417.47 nm after 3 h. With the powder XRD model, the mean crystallite dimension of nanoparticles was determined as 12.17 mm with a cubic structure. According to the TEM results, the dimensions of the obtained silver nanoparticles were found to be 6.11–9.66 nm. The ZP of the electric charge on the surface of AgNPs was measured as −19.6 mV. The inhibition effect of AgNPs on food pathogen strains and yeast was determined with the minimum inhibition concentration (MIC) method. AgNPs demonstrated highly effective inhibition at low concentrations especially against the growth of *B. subtilis* (0.0625) and *S. aureus* (0.125) strains. The cytotoxic effects of silver nanoparticles on cancerous cell lines (CaCo-2, U118, Sk-ov-3) and healthy cell lines (HDF) were revealed. Despite the increase of AgNPs used against cancerous and healthy cell lines, no significant decrease in the percentage of viability was detected.

## Introduction

Nanotechnology is revealing new perspectives for the diagnosis and cure of numerous deadly autoimmune and chronic disorders like cancer (Kafshdooz et al., 2018; Yadi et al., 2018). Nanoparticles have become the main subject of scientific works in the last few decades because of their diverse properties, like different catalytic behaviors, chemical stability, and electric conductivity (Patra and Baek, 2016). Nanoparticles have become an indispensable source of biological research due to their structural and dimensional similarities to biological molecules. Nanoparticles are considered antimicrobial agents because they show good antibacterial properties resulting from their extensive surface area and volume that provides desired contact with the bacterial cell (Kumar et al., 2016). These properties allow nanoparticles to be used in diagnostic, cell labeling, biomarker, drug delivery, cancer therapy, and water purification applications (Mousavi et al., 2018; Kumari et al., 2019; Kowsalya et al., 2021).

In recent years to examine the morphological properties of nanoparticles, laser CVD, physical adsorption, and emulsion polymerization techniques are commonly being used. However, these technologies require the usage of stabilizing/reducing harmful chemicals or non-biologically degradable agents (Jayaprakash et al., 2017). For this reason, it is preferred to produce nanoparticles with fast, low-cost “green synthesis” procedures that do not use toxic solvents or pollute the environment, instead of current traditional methods (Hussain et al., 2016; Jayaprakash et al., 2017; Bandeira et al., 2020). Living organisms in nature can convert metal salts into nanoparticles by reducing them. In this context, scientific studies have focused on synthesizing these nanomaterials from non-artificial sources like plants (Aktepe and Baran, 2021), bacteria (Javaid et al., 2018), fungi (Molnár et al., 2018), algae (Parial et al., 2012), seaweeds (Chellapandian et al., 2019), and viruses (Mohmed et al., 2017).

In many nanoparticle studies, gold (Au) (Hatipoğlu, 2021), silver (Ag) (Baran, 2019; Umaz et al., 2019; Baran et al., 2021b), zinc (Zn) (Jayappa et al., 2020), nickel (Ni) (Din et al., 2018), iron (Fe) (Devatha et al., 2016), platinum (Pt) (Ramkumar et al., 2017b), selenium (Se) (Abu-Elghait et al., 2021), titanium (Ti), and palladium (Pd) (Gioria et al., 2020) are frequently used metals. Especially silver (Ag) is known to be an important metal suppressing the growth of bacteria. The Ag ion can prevent cell division and DNA replication (Ramya and Subapriya, 2012). Owing to their small dimensions, silver nanoparticles (AgNPs) bind to cell membrane proteins and catalyze the formation of reactive oxygen species (ROS) in bacterial cells. Thus, they cause cell death due to oxidative stress (Hoseinnejad et al., 2018; Alkhalaf et al., 2020; Hasanzadeh et al., 2021).

The most important advantage of choosing plants as a resource in the biological synthesis of nanoparticles (NPs) is that they contain many naturally occurring reducing agents such as flavonoids, reductases, phenolic acids, and dehydrogenases, which have a key role in the synthesis of magnetic nanoparticles (MNPs) (Shumail et al., 2021).

**FIGURE 1:**
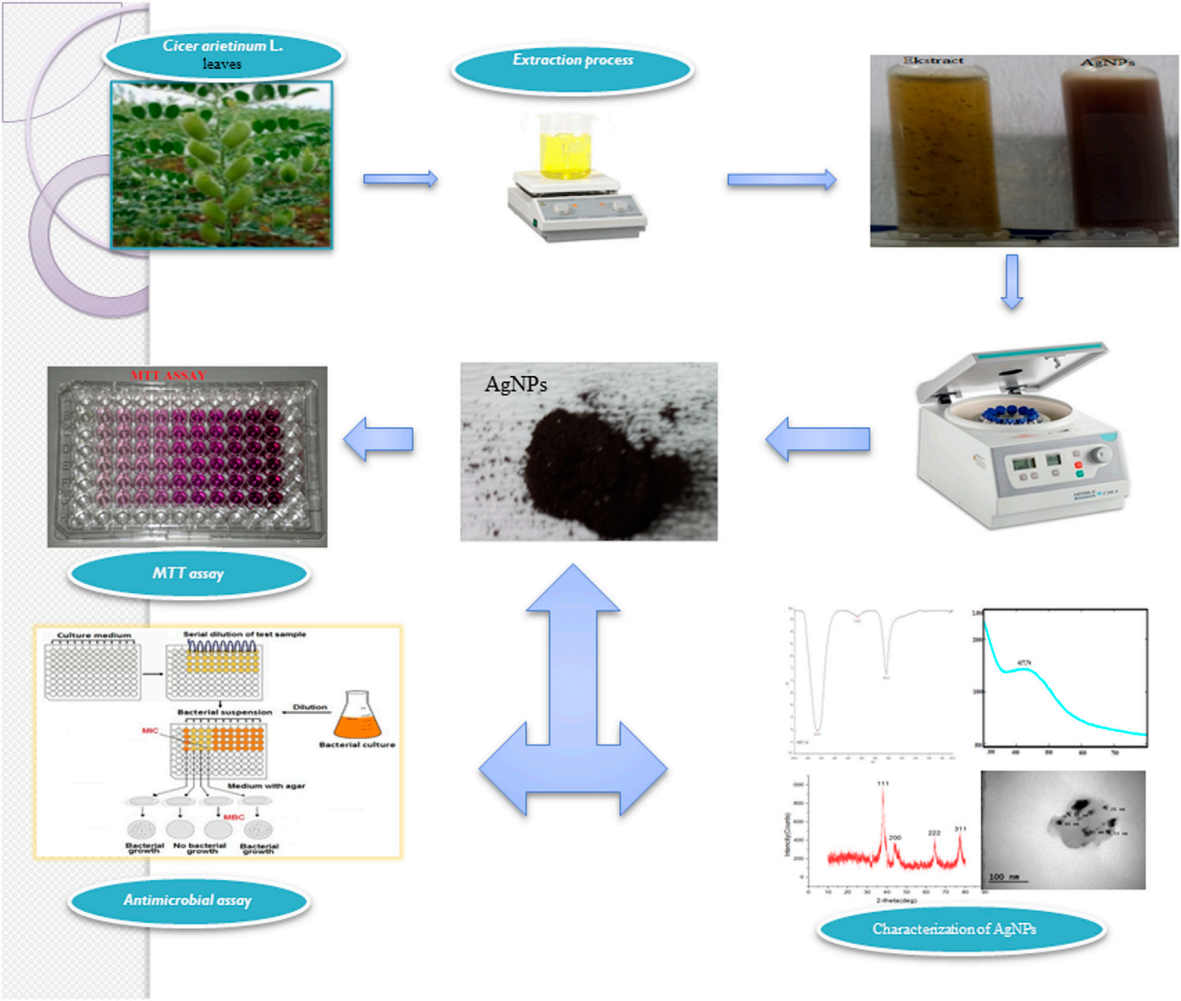
Graphical illustration of the study.

In this study considering the properties of plants, the synthesis and stabilization of silver nanoparticles were achieved by reducing Ag metal salt by using chickpea (*Cicer arietinum* L.) (CA) leaf extract. Plant-based synthesized AgNPs were investigated for their effectiveness against pathogens, microorganisms, and cancerous and healthy cell lines (Figure 1).

## Materials and Methods

### Materials

In the study, CA leaves obtained from Büyük Çelikli Village of Sur County of Diyarbakır were used. AgNO3 (99.8% purity), colistin, vancomycin, and fluconazole were commercially purchased from Sigma Aldrich. E. coli ATCC 25922, *P. aeruginosa* ATCC27853, *B. subtilis* ATCC 11774, *S. aureus* ATCC 29213, and C. albicans were used to test the antimicrobial activities of AgNPs. Cytotoxicity tests (MTT) related to cell lines (CaCo-2/human colon epidermal adenocarcinoma; U118 MG/human brain glioma cells; SK-OV-3/human ovarian cancer cell line; HDF/human dermal fibroblasts) were performed in the Dicle University Central Research Laboratory.

### Herbal Extraction Process

Green leaves of CA were washed with deionized distilled water to remove residues and dehydrated at 25 ± 2°C. A total of 250 g of ground plant material was mixed with deionized pure water (500 ml) and boiled in a flask. After boiling, the cooled extract was filtered with a membrane filter (0.45 μm).

### Plant-Based Synthesis of Silver Nanoparticles

Firstly, an aqueous solution of 5 mM AgNO_3_ with solid AgNO_3_ was prepared. The CA extracts (500 ml) and 100 ml of AgNO3 were allowed to react in a glass vessel (1:5 ratio) at room temperature. Maximum absorbance of biologically synthesized AgNPs was determined by wavelength scanning (UV-vis spectroscopy) at various time periods (15, 30, 45, 60, 120, and 180 min) depending on the color change. At the end of the synthesis, the solution, which became a dark color depending on time, was subjected to centrifugation (6000 rpm, 20 min). The purpose of this process is to separate the synthesized nanoparticles from plant residues. The solid fraction obtained at the end of centrifugation was washed several times with distilled water and the resulting residue (AgNPs) was dried in an oven at 60°C for 72 h.

### Instrumentation

The maximum absorbance of synthesized AgNPs was measured at the 300–800 nm wavelength range with a spectrophotometer (Agilent CARY 60). Size, morphology, crystal structure, surface distribution, and zeta potential (ZP) values of AgNPs were revealed by scanning electron microscopy (SEM) (EVO 40 LEQ), transmission electron microscopy (TEM) (Quanta), field emission scanning electron microscopy (FE-SEM) (Quanta FEG240), electron dispersive X-ray (EDX) (Quanta FEG 240), X-ray diffraction analysis (XRD) (Rad B-DMAX II), and Zetasizer (Malvern Ins. Ltd.). The crystal dimension of AgNPs was calculated according to the D = Kλ/(β cosθ) equation (Asadi et al., 2018; Baran et al., 2018). In addition, Fourier transform infrared spectroscopy attenuated total reflectance (FT-IR ATR) was used to identify the functional groups present in the CA extract, and the functional groups responsible for the reduction at the end of the reaction test analysis conditions of used instruments are given in Table 1.

**TABLE 1:** Instrument conditions.

Instrument	Condition
SEM-EDX (EVO 40 LEQ)	Mag: 500–60.00 K X; EHT: 20.00 kV; WD: 11–12 mm; Signal A: SE1
TEM (Quanta)	1–100 nm
XRD (Rad B-DMAX II)	Dedector: SC-70; Solid phase; 2-theta (deg): 37.96; FWHM (deg): 1.17; Count (deg): 184; X-Ray: 40 kV, 15 mA
Zeta-sizer (Malvern Ins.Ltd.)	Zeta Deviation (mV): 5.81; Viscosity (cP): 0.8872; Conductivity (mS/cm): 0.00843; Dispersant Dielectric Constant: 78.5; Temperature (°C): 25; Count Rate (kcps): 93.3
FT-IR ATR (Perkine Elmer ONE)	Strong Ratio Spectrum Magnitude Universal Atr Double

### Antimicrobial Activities of Silver Nanoparticles

Growth inhibition of plant-based AgNPs on Gram-positive (*B. subtilis*; *S. aureus*) and Gram-negative (*E. coli*; *P. aeruginosa*) strains and yeast (*C. albicans*) was determined using a 96-well microplate with minimum inhibition concentration (MIC) method. Mueller Hinton broth and cell culture growth medium (Roswell Park Memorial Institute medium/ RPMI) were added to the wells for the growth of bacteria and yeast. The AgNP solution was added to the wells with the culture medium and microorganisms to determine the MIC value. Firstly, 100 µL of mixed culture medium was taken from the wells each time and transferred to the next well. Then the microorganism solutions adjusted according to the 0.5 McFarland standard were added to the microplates and incubated at 37°C/24 h. The minimum concentration without growth after incubation was determined as the MIC value (Baran et al., 2020). Commercially purchased standard antibiotics (colistin, vancomycin, and fluconazole) and 1 mM AgNO_3_ solution were used to compare the growth inhibitory activities of AgNPs on pathogen microorganisms.

### Evaluation of Viability Suppressor Activities of AgNPs by the MTT Method on Cell Line Seeding in a 96-Well Plate

The MTT method was performed to determine the plant-based AgNP ratio of cytotoxicity (viability suppressor) on cancerous and healthy cell cultures. T-75 T-flasks were used to prepare the culture medium. CaCo-2, HDF, and U118 cell lines were cultivated in DMEM solution. The human ovarian cancer cell line (SK-OV-3) was incubated in RPMI solution. Prepared cultures were incubated at 5% CO2, 37°C, and 95% air and humidity conditions. When the cells reached about 80% confluency in the hemocytometer measurement, cell cultures were suspended at different concentrations and transferred to microplates (96-well) for incubation (overnight). At the end of the period, the cultured cell lines were treated with nanoparticles at different concentrations (25, 50, 100, and 200 μg/ml) and incubated for 2 days. In the next step, the MTT solution was transferred to the microplate wells and incubated for 3 h, and then DMSO was added and kept at room temperature for 0.25 h. The absorbance (540 nm) of the microplates was measured with MultiScan Go (Thermo).

By using the below formula, the percentage viability of the cell lines was calculated.

% viability = U/C*100 (Vickers, 2017).

U: Absorbance of cells treated with AgNPs.

C: The absorbance values of control cells.

## Results and Discussion

### UV-Visible Spectroscopic *Analysis*

**FIGURE 2:**
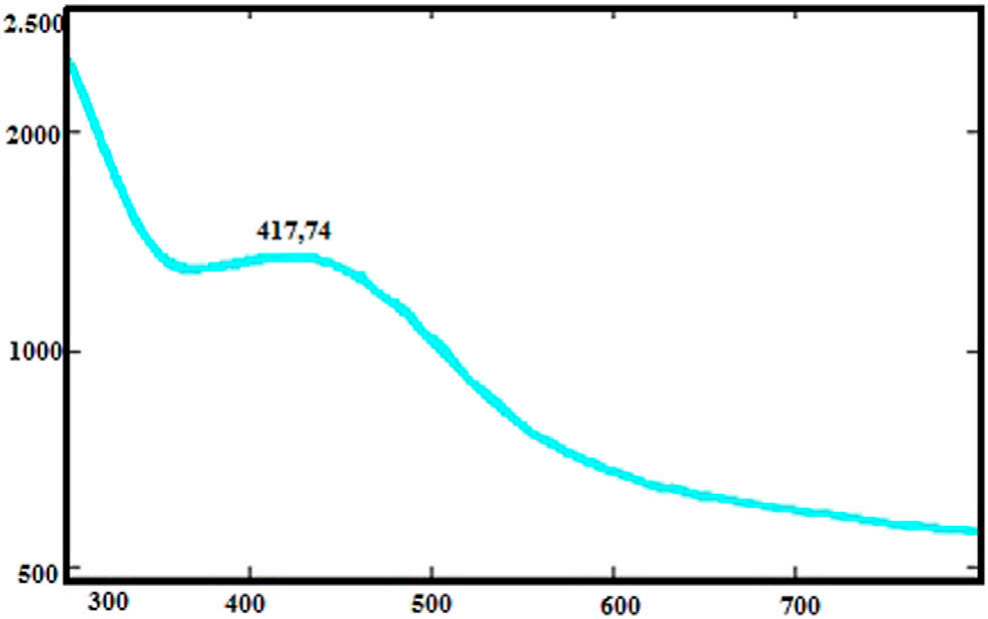
UV-vis absorption spectra of CA-AgNPs.

The color change was observed after the CA leaf extracts with AgNO3 solution were left to react in a container. The UV-vis spectrum of AgNPs appeared to change from light green to purple (Figure 2). Because of the surface plasmon resonance, AgNPs gave a peak at a specific absorbance value of about 417.47 nm. Similarly, some researchers reported that the absorption spectrum of AgNPs is between 425–461 nm (Udayasoorian et al., 2011).

### Evaluation of SEM and TEM Data

**FIGURE 3:**
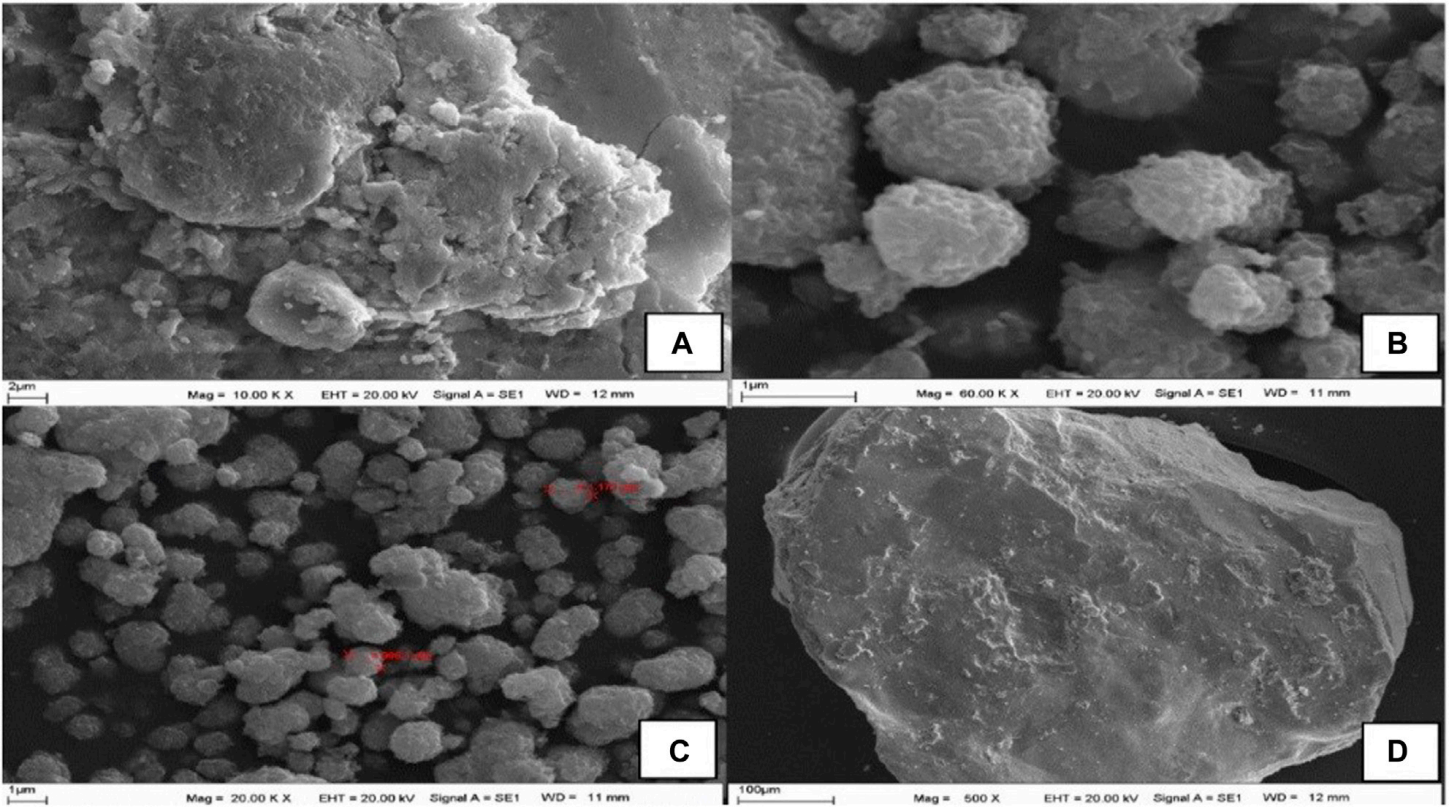
**(A–D)** SEM images of CA-AgNPs in different scanning areas.

**FIGURE 4:**
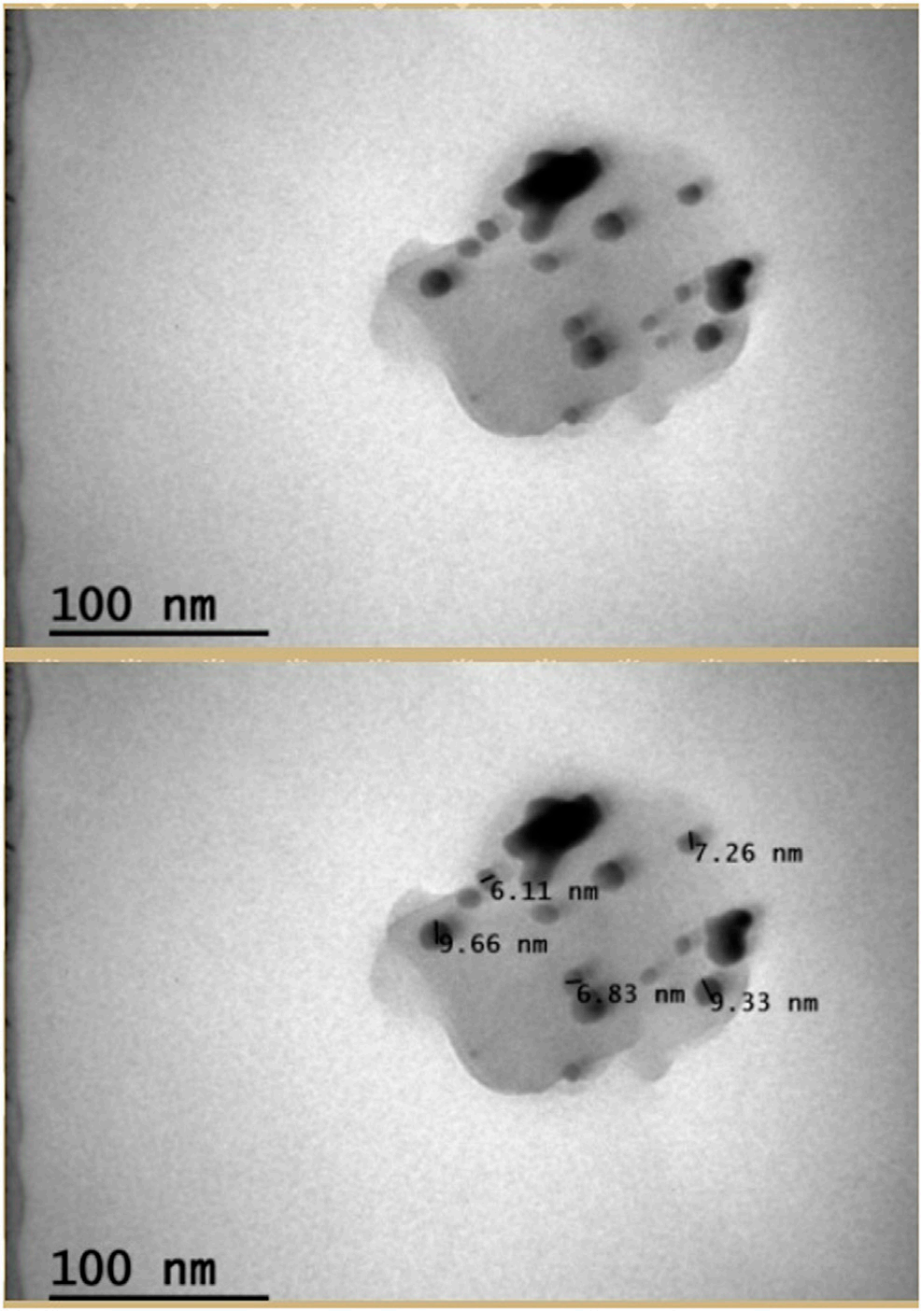
TEM results of CA-AgNPs.

SEM, FE-SEM, and TEM images of synthesized AgNPs are given in Figures 3, 4. According to these results, it was seen that the nanomaterial was mostly spherical, nano-sized, and in clusters that were not in direct contact with each other. This indicates the stabilization of the AgNPs. It was reported that AgNPs have a spherical morphology and nano-dimensions in similar studies (Ramkumar et al., 2017a; Pallela et al., 2018). The biosynthesized nanoparticles are expected to have stronger antimicrobial activity, on account of their relatively small size. In the particle measurement done with TEM, it was seen that the sizes of AgNPs were approximately between 6.11–9.66 nm and the average size was approximately 7.83 nm (Figure 4). In some studies, using different materials, the sizes of AgNPs were reported to be between 2–95 nm (Nguyen et al., 2018; Behravan et al., 2019).

### Evaluation of FT-IR Analysis Data

**FIGURE 5:**
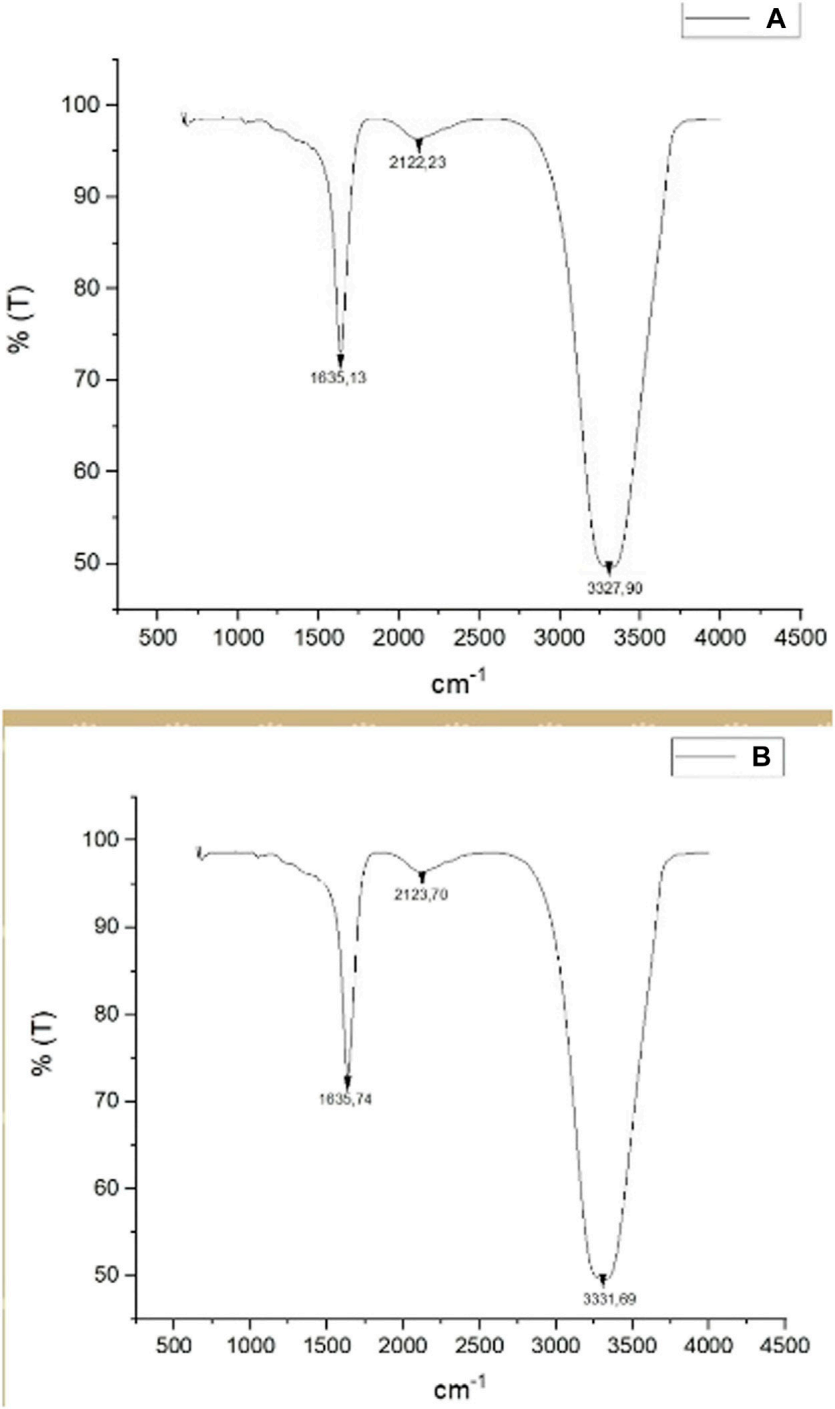
**(A)** FT-IR spectra data of CA leaf extract. **(B)** FT-IR spectra data of synthesized CA-AgNPs.

The FT-IR spectroscopy analysis determined the functional groups involved in the plant-derived reduction. The frequency of all stretch in the range of 4500–500 cm^−1^ was recorded with four scans at 1 cm^−1^ resolution. Figure 4 shows a comparison of FT-IR spectra for the aqueous CA extract (Figure 5A) and synthesized AgNPs (Figure 5B). When the biomolecules involved in reduction during the formation of AgNPs were examined (Figures 5A,B), the absorption peak at 1635 cm^−1^ corresponded to the C=O stretching vibration, indicating the presence of amide. Because of the phenolic compounds in the CA leaf extract, it can be concluded that the absorption peak at 2122 cm^−1^ belongs to alkyne (C≡C) groups while the absorption peak at 3331 cm-1 belongs to O-H and N-H stretching (Atalar et al., 2021). Presumably, these determined functional groups are responsible for the reduction of metal ions (Mandal et al., 2015).

### Evaluation of EDX Analysis Data

**FIGURE 6:**
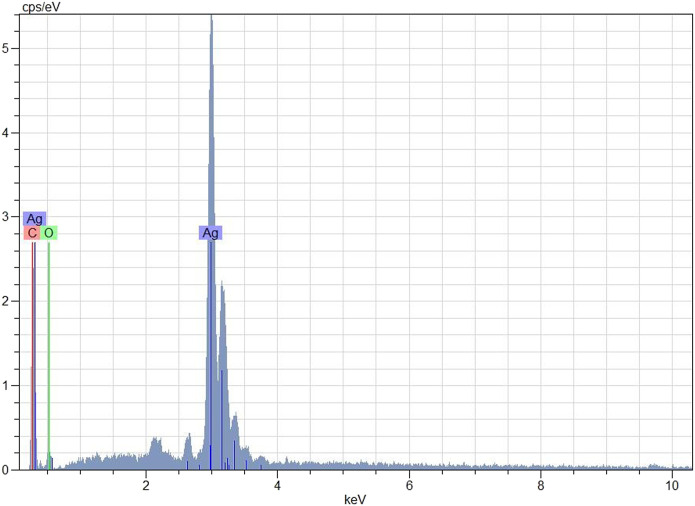
Elemental composition of AgNPs with EDX analysis.

According to the EDX profile (Figure 6), it was confirmed that the biosynthesized nanoparticles had silver in their composition. It was also seen that the elemental composition of silver was high (Figure 6). AgNPs showed a typical optical absorption peak at about 3 KeV owing to the surface plasmon resonance (SPR). It can be said that the other emerging peaks are because of phytochemicals attached to the surface of AgNPs in the CA leaf pulp (Punuri et al., 2012). Khamhaengpol and Siri (2017) and Dada et al. (2019) also revealed the EDX silver peaks in their work.

### XRD Analysis

**FIGURE 7:**
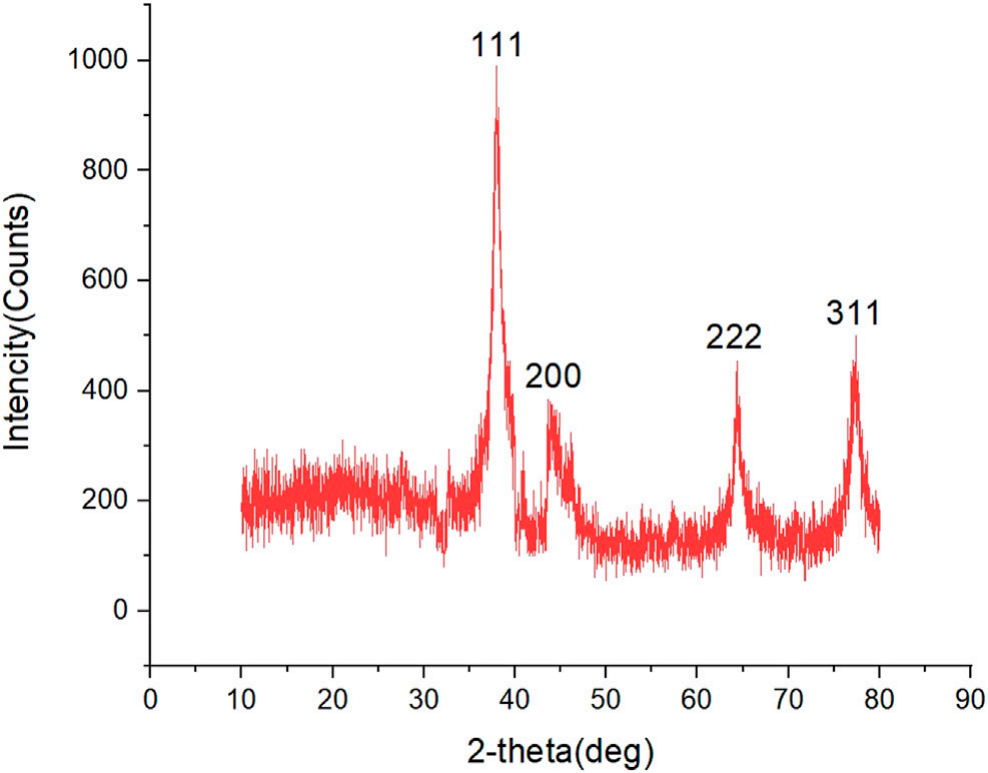
XRD patterns of biosynthesized AgNPs.

The XRD spectrum model for the synthesized AgNPs is shown in Figure 7. In XRD analysis results, peaks of 111^o^, 200^o^, 220^o^, and 311^o^, which coincide with 37.96, 44.29, 64.32, and 77.33, respectively at 2θ, were sharp peaks representing the spherical crystal structure of silver. The peaks indicated that the AgNPs were cubic in structure. It has been reported in many studies that these peaks belong to silver (Huang et al., 2019; Keskin et al., 2021). The highest peak, 37.96, was taken as the peak angle. The size of the nanomaterials was calculated as approximately 12.17 nm according to the below equation ((Baran et al., 2021a).

D=Kλ/(β⁡cos⁡θ)

In the equation, D = the size of the particle, K = the constant value (0.89), λ = the wavelength value of XRD (1.5418 Å), β = the FWHM value of the high peak, and cosθ = the Braggθ angle of the high peak.

### Evaluation of Zeta Potential Analysis Data

**FIGURE 8:**
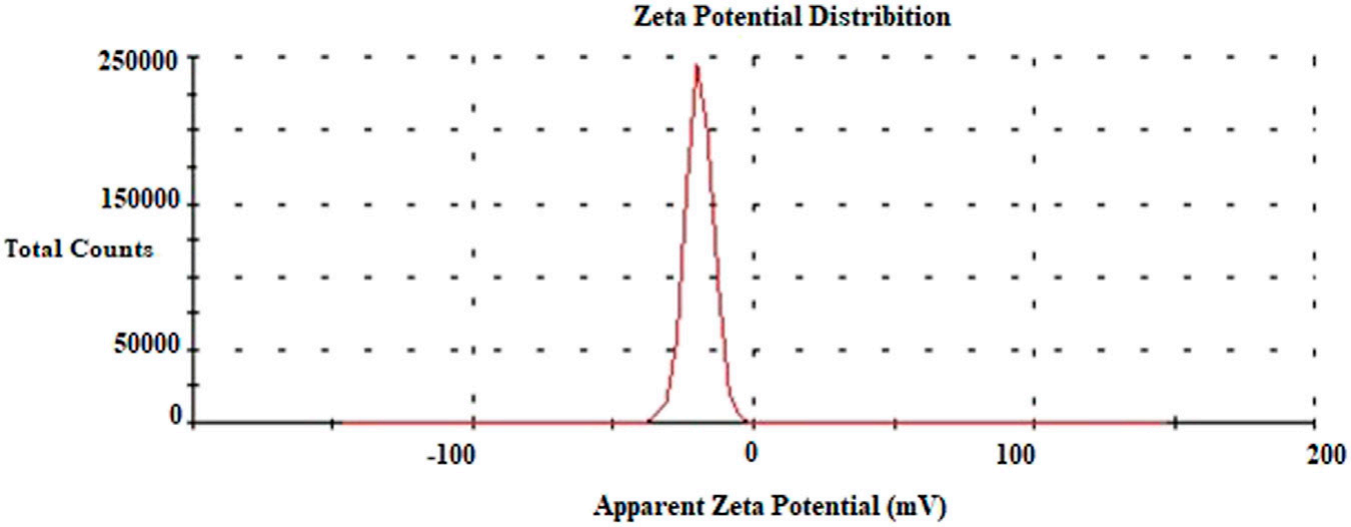
Zeta potential data of AgNPs.

The zeta potential analysis gives the electric charge on the surface of the surrounded material. The high negative value of the zeta potential prevents the particles from sticking together or clumping together. This indicates the stability of the AgNP colloid. On the other hand, nanoparticles with a significantly lower negative charge can enter the cell more easily (58–60). The zeta potential of the biosynthesized AgNPs was found to be −19.6 mV (Figure 8). This value indicated that the AgNPs were stable and uniformly distributed. The different zeta potential values of AgNPs synthesized from various materials have been reported previously (Ferreyra Maillard et al., 2018; Amer et al., 2020).

### Evaluation of Antimicrobial Properties of AgNPs

The antimicrobial effects of AgNPs have become more important due to microorganisms that cause disease in humans developing resistance to conventional antibiotics. *S. aureus*, *B. subtillis, E. coli*, and *P. aeruginosa* strains and *C. albicans* yeast are pathogenic microorganisms frequently encountered in food-borne diseases (Yang et al., 2017; Mostafa et al., 2018). It was determined that the biosynthesized AgNPs significantly inhibited the growth of these microorganisms even at low concentrations (Table 2). It was observed that AgNPs strongly inhibited the growth of *S. aureus* and *B. subtilis* when compared to other microorganisms. Since silver has a strong tendency to interact with phosphorus and sulfur atoms in the bacterial cell wall, it interacts with the thiol and phosphorus groups in the bacterial cell membrane, thereby disrupting the bacterial respiration process. This causes the death of bacteria (Hamouda and Baker Jr, 2000). On the other hand, since the cell wall of Gram-positive bacteria has a hard polysaccharide layer, the transition to the Gram-positive bacterial wall is more difficult when compared to Gram-negative bacteria. Therefore, the inhibitory activity of AgNPs in Gram-positive bacteria is stronger than in Gram-negative bacteria (Tamboli and Lee, 2013). Thuc et al. (2016) reported that Gram-positive *S. aureus* has approximately 2–3 times higher resistance to AgNPs than Gram-negative *E. coli* and *P. aeruginosa* (Thuc et al., 2016). These effective inhibitory activities of AgNPs on different bacteria strains and yeasts were also reported by many researchers (Niknejad et al., 2015; Aygün et al., 2020).

**TABLE 2:** MIC results of AgNPs, AgNO_3_, and standard antibiotics (μg/ml).

Microorganisms	AgNPs	AgNO_3_	Antibiotics^a^
*B. subtilis* (Gram-positive)	0.0625	1.32	1
*S. aureus* (Gram-positive)	0.125	2.65	2
*P. aeruginosa* (Gram-negative)	1.0	1.32	4
*E. coli* (Gram-negative)	1.0	0.66	2
*C. albicans* (yeast)	0.5	0.66	2

a Colistin: Gram-negative bacteria; Vancomycin: Gram-positive bacteria; Fluconazole: *Candida albicans*.

### Evaluation of Cytotoxic Activities of AgNPs

**FIGURE 9:**
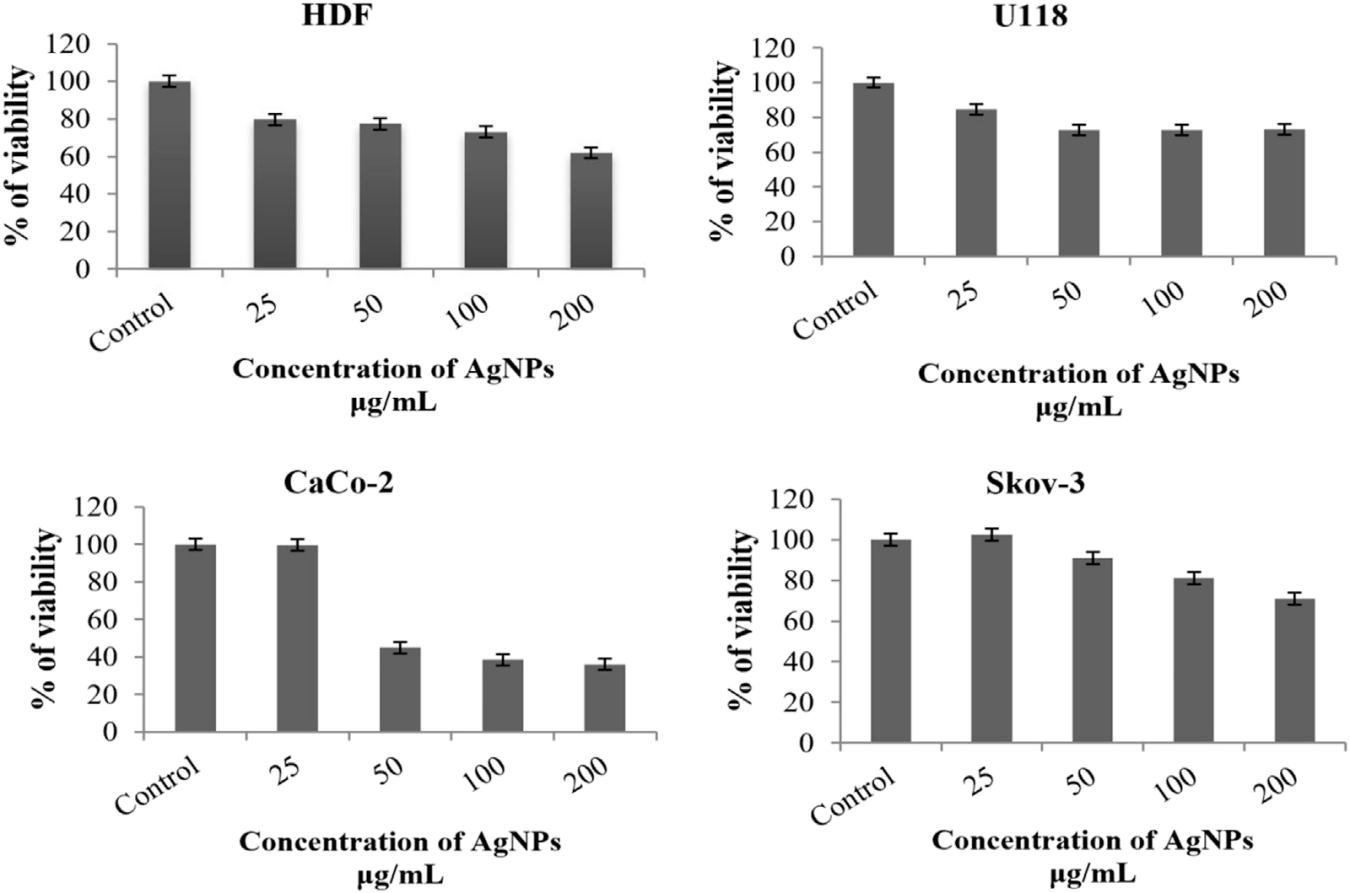
Evaluation of the percentage viability rates as a result of the cytotoxic effect of AgNPs 2 days after combining them with CaCo-2, U118, HDF, and Sk-ov-3 cell lines.

AgNPs obtained by biosynthesis of chickpea leaf extract were applied to healthy cells (HDF) and three different cancer cell lines (CaCo-2, U118, and Sk-ov 3), and the results obtained after 48 h are shown in Table 3 and Figure 9. According to these results, it was seen that there was no toxic effect in HDF with a survival rate of 79.70% at a 25 μg/ml concentration. It was determined that the most suppressed concentration of viability was on CaCo-2 cells at 200 mg/ml (Table 3). Despite the increase in the concentration of AgNPs in other cancer cell lines, the increase in the percentage of viability can be explained by the proliferative properties of AgNPs for these cells (Morais et al., 2020).

**TABLE 3:** The percentage viability rates of the cell lines suppressed with AgNPs.

Cell line	Concentration µg/mL			
	25	50	100	200
U118	84.53117	72.77605	72.73556	73.18908
CaCo-2	99.74733	44.98866	38.54875	36.04794
Sk**-**ov-3	102.5666	91.0701	80.9948	70.88769
HDF	79.70489	77.31011	73.07289	61.86905

It is known that AgNPs show strong oxidative properties (Wongpreecha et al., 2018). AgNPs tend to settle in biomolecules such as cell membranes and nuclei. They exert a toxic effect by stimulating apoptosis with an increase in ROS after localization (Gliga et al., 2014; Morais et al., 2020). The concentration, exposure time, shape, size, charge, degree of deposition, and chemistry of the surface composition can have a significant impact on the toxicity of AgNPs (Swamy et al., 2015). In studies conducted to examine the toxic effects of AgNPs on CaCo-2 cells, it was reported that concentrations above 3.75–5.5 μg/ml showed toxic effects (Zein et al., 2020). Inhibitory concentrations in Sk-ov-3 cells were reported to be 9.4 μg/ml (Fahrenholtz et al., 2017) and 29.36 μg/ml (Noor et al., 2021). Zhang et al. (2010) reported that the 100 μg/ml concentration of silver nanoparticles was toxic on cell viability on HDF cell lines (Zhang et al., 2010).

## Conclusion

In this study, green synthesis of silver nanoparticles (AgNPs) was carried out using *Cicer arietinum* leaf extract in a low-cost, environmentally friendly, simple, and fast method. No toxic or hazardous substances were used in the biosynthesis. The rapid and green synthesis of CA-AgNPs was successfully completed using the available phytochemicals in *Cicer arietinum* leaf extract as reducing agents. SEM and TEM images showed that spherically symmetrical plant-based AgNPs were formed due to their high stability. UV-vis absorption, XRD, and EDX analyses confirmed the synthesis of silver nanoparticles. Various microscopic analyses indicated that AgNPs had mostly spherical morphology with an average size of about 7.83 nm. The obtained analysis data showed that the smaller the size of the nanoparticles, the greater their antimicrobial activity, and the obtained AgNPs had strong antibacterial and anticandidal activity even at very low concentrations. The cytotoxic activities of CA-AgNPs were evaluated by the MTT method. A 25 μg/ml concentration of CA-AgNPs suppressed healthy cells by 20% and suppressed the viability of cancer cell lines by 1–15%. As the concentration increased, the suppression rate in cell lines other than U118 also increased. It was determined that silver nanoparticles synthesized using plant material had a high suppressive effect on the viability of CaCo-2 cells in parallel with the increase in concentration. It is known that NPs can be used in many commercial products for biological and medical applications. According to the results obtained, it is thought that CA-AgNPs can be used effectively as antimicrobial and anticancer agents in the food industry and medical applications.

## Data Availability

The original contributions presented in the study are included in the article/Supplementary Material, further inquiries can be directed to the corresponding authors.
